# Trends in Haptic Communication of Human-Human Dyads: Toward Natural Human-Robot Co-manipulation

**DOI:** 10.3389/fnbot.2021.626074

**Published:** 2021-02-17

**Authors:** Spencer W. Jensen, John L. Salmon, Marc D. Killpack

**Affiliations:** Robotics and Dynamics Laboratory, Brigham Young University, Mechanical Engineering, Provo, UT, United States

**Keywords:** haptic communication, visual communication, dyad, physical human-robot interaction (pHRI), human-human interaction (HHI), interaction force/torque, co-manipulation, robotics

## Abstract

In this paper, we analyze and report on observable trends in human-human dyads performing collaborative manipulation (co-manipulation) tasks with an extended object (object with significant length). We present a detailed analysis relating trends in interaction forces and torques with other metrics and propose that these trends could provide a way of improving communication and efficiency for human-robot dyads. We find that the motion of the co-manipulated object has a measurable oscillatory component. We confirm that haptic feedback alone represents a sufficient communication channel for co-manipulation tasks, however we find that the loss of visual and auditory channels has a significant effect on interaction torque and velocity. The main objective of this paper is to lay the essential groundwork in defining principles of co-manipulation between human dyads. We propose that these principles could enable effective and intuitive human-robot collaborative manipulation in future co-manipulation research.

## 1. Introduction

### 1.1. Background

Basic principles of human-human haptic communication are largely still a mystery to researchers even though humans communicate through haptics every day with activities such as holding hands on a walk, dancing, or moving an object too large or heavy for one person to carry. The language of haptic communication is complicated and can convey many simple intentions such as a desired speed or direction as well as emotional or physiological states such as fatigue or stress. Throughout this work, important preliminary principles are developed about haptic communication and how they specifically relate to carrying an object of significant mass and length in six degrees of freedom (DoF).

The development of robots that are capable of physical Human-Robot Interaction (pHRI) is becoming more of a focus as industries seek to combine the intelligence and adaptability of humans with the precision and potential reliability of robots. We refer to collaborative manipulation of a single object between multiple agents (robots, humans, or a mixture of both) as “co-manipulation.” One major and straightforward application for co-manipulation is a search-and-rescue robot that could help with carrying a stretcher or removal of rubble. Search-and-rescue operations are often performed in harsh or unfriendly environments and a robot could decrease the number of people in harm's way as well as provide a level of care to the patient that would otherwise be impossible. Understanding human-human interaction (HHI) will be an integral part of that process if we expect humans to intuitively interact with robots in these scenarios. Robots are currently able to do extraordinary things from creating millions of cars in a manufacturing plant to performing delicate surgeries. In most of these tasks, humans are not allowed in the same space as the robots and the robots are pre-programmed or teleoperated. We propose that for pHRI to become ubiquitous, robots need to (1) move in ways that are predictable for human partners, (2) be able to communicate naturally with humans, and (3) be able to work with novice users. We seek to fulfill these needs by studying human-human interaction.

While haptic feedback has often been implemented as the only means of communication in pHRI co-manipulation tasks (Lecours et al., 2012; Whitsell and Artemiadis, 2017; Jaroonsorn et al., 2019), the effect of limiting communication to only haptic feedback for six DoF tasks has not been investigated in depth. Because of advances in computer vision, eye gaze detection, and voice-to-text abilities, these methods of communication are now possible to include inside the programming loop in future work. In this study, we quantify to what extent removing both visual and auditory cues will hamper human-human co-manipulation. The next several paragraphs illustrate the current state of pHRI controllers, previous research in human-human co-manipulation, and important achievements in research relating to haptic and non-haptic human-human communication.

### 1.2. Related Work

For many years impedance control has been the dominant control algorithm for pHRI. Ikeura et al. (2002) implemented variable impedance control for lifting an object and proved that it was effective in completing the task. Several researchers have continued similar development using algorithms related to impedance control, such as Gopinathan et al. (2017), who found that adaptive stiffness control increased performance over fixed stiffness and gravity compensated control. One of the problems with impedance control is that it usually requires off-line tuning. Ranatunga et al. (2015) built a cascading loop controller that did not require prior off-line tuning. They changed the impedance of the robot according to the prediction of motion of the human. The controller worked well, however, the experiments were only carried out with basic linear arm movements. Lecours et al. (2012) took variable impedance control and extended it to four degrees of freedom. This work showed that humans can successfully indicate their desired direction with only haptics. Li et al. (2016) built a controller based on game theory and the human's input force on the robot's end-effector and showed that it was functional. Whitsell and Artemiadis (2017) developed a successful controller for six DoF pHRI. However, their research only involved small arm movements and the controller essentially held four DoF stable while allowing the human to change two DoF at a time dependent on how much force was applied. Jaroonsorn et al. (2019) developed a novel pHRI controller that combined fuzzy logic and proportional-integral control. This controller achieved its functional goal, however, to our knowledge there was no qualitative data presented that related the legibility and predictability of the robot for the human in this study, where legibility and predictability are defined in Dragan et al. (2013).

One large problem in pHRI is the difficulty for a follower to haptically discern if the leader wants the follower to move an object in a lateral collaborative motion (translation) or if the leader wants to cause the object to pivot about the follower (rotation). Dumora et al. (2012) investigated this problem and found that haptic communication for rotation about the follower and for lateral translation (leader and follower move sideways together) was not significantly different. Arai et al. (2000) implemented a virtual non-holonomic constraint to overcome this problem. However, it required the human to perform extra actions similar to using a wheelbarrow.

Minimum jerk (MJ) trajectories have been introduced as a desirable metric for movement (Burdet and Milner, 1998). In our own prior work, we found that humans exhibit behavior similar to a minimum jerk trajectory while moving laterally (Mielke et al., 2017). However, Parker and Croft (2011) found that humans did not respond well to MJ movements faster than 0.5 Hz. Later, Parker and Croft (2012), in studying a lifting task, found that humans reacted better to a second-order trajectory than a minimum jerk trajectory. In a role exchange pHRI study performed by Kucukyilmaz et al. (2013) more than half of the participants interacting with the robot verbally stated that the robot was too smooth to be a human. Ikeura and Inooka (1995) studied HHI to predict parameters for impedance control for a one degree of freedom task. In their study, they instituted a leader and follower setup where only the leader knew where the goal location was defined. Rahman et al. (2000) hypothesized that the leader was responsible for the external force and the follower was responsible for internal forces and built a successful impedance controller based on this assumption. A significant drawback of this study was the low dimensionality of the task.

While studying HHI, Reed K. et al. (2006) found that when two people co-manipulate a single object they are significantly faster than a single person. Additionally, a dyad exerts twice as much force as a single person with most of their effort being canceled out by the other person. They also found that a significant portion of the dyads in their study ended up in a specialized relationship where one person was responsible for acceleration and the other deceleration. They hypothesized that this allowed for the dyad to come closer to an optimal bang-bang control approach. Reed K. B. et al. (2006) cited Reinkensmeyer et al. (1992) who found a similar result when a single person moved a pencil with two hands. Finally, in Reed and Peshkin (2008) they developed a robot controller that took over the role of accelerating the object as seen in human-human studies. However, this controller lost the performance gain of a dyad and ended up being quite similar in time to an individual performing the task alone.

While exploring dominance and haptic decision making in HHI, Groten et al. (2009) defined interaction force as the counteracted applied forces that did not create movement of the object. In a study of several dyads performing forward and backward dance movements, Sawers et al. (2017) found that expert dyads applied significantly greater interaction forces than novice dyads, suggesting that the extra force increased communication levels in the partnership. Sylos-Labini et al. (2018) found that dyads walking side-by-side also exhibited significant interaction forces and hypothesized that this interaction force helped communicate synchrony. Lanini et al. (2017) performed a human-human experiment where a human dyad carried a stretcher-like object forward and backward and found that the human dyad would often fall into synchronous steps. They assumed that interaction forces between the carriers enabled them to stay in synchrony.

While haptic feedback is indeed important, the importance of other communication pathways is also investigated in this paper. Li and Zhang (2017) proved that important intention communication can come from eye gaze by asking participants to communicate a task through gaze intention. Implementing an SVM classifier they were able to accurately predict the communicated task. Eils et al. (2017) found that visual cues significantly improved the performance of a dyad in a whole-body balancing task. Eils et al. (2017), however, did not provide a direct extension to six DoF co-manipulation tasks. Mojtahedi et al. (2017) performed a study on visual and haptic communication between a dyad with a simple arm movement task and found that visual feedback was not significantly important. It is worth noting that they found that the number of trials or repetitions was the second highest factor in determining the dyads' performance. Parker and Croft (2011) ran a lifting and holding experiment with a human and robot with the human alternately blind-folded and not blind-folded. They found that when the human was blind-folded they could not accurately level the load, however the participants' reaction time and stability remained constant. They also found that a tension load was applied to the object by the human and that the tension increased on average when the blindfold was applied from 4.8 to 8.8 N. They hypothesized that the increased tension helped the human partner follow the motion of the robot.

### 1.3. Study Purpose

Altogether the works presented above represent huge leaps in the understanding of HHI and in the development of successful pHRI controllers. However, relatively little research has gone into building robot controllers for pHRI from HHI. This becomes a problem since the design of the controller predominantly dictates the interaction with the human partner and although successful pHRI controllers exist, the success may be a product of the adaptability of humans instead of reacting as they would nominally (Sawers and Ting, 2014). This leads to a large gap in the literature where haptic interactions are inadequately understood in HHI to allow development of robot controllers to mimic that interaction.

There are two significant gaps in current pHRI and HHI literature: (1) most of the research was conducted with humans interacting with the end effector of the robot instead of communicating through an object of significant mass and length, and (2) the potential lack of extensibility to six DoF tasks. We hypothesize that a six DoF co-manipulation task with an object of considerable weight and length may significantly change the behavior of previously studied human dyads and therefore change the requirements for a robotic controller that would attempt such a task in human-robot interaction. We also hypothesize that interaction forces will be a necessary part of a good pHRI controller because of its presence in previous HHI studies. Furthermore, we propose that our findings could also apply to tasks where the dyad naturally suffers from visual or auditory impairments such as: carrying a large object or moving around in a smoky or noisy environment. By studying human-human interaction (HHI), we provide empirical data to help design a robot controller that will feel legible and predictable to humans. In the rest of this paper, we lay out our methods and procedures for experimentation, the analysis and results of our findings and discuss the implications and limitations of our current study.

## 2. Materials and Methods

### 2.1. Experiment Design

To better understand human-human haptic communication, we devised an experiment where a dyad completed a six DoF set of whole-body co-manipulation trials with an object of significant length and mass. The experiment was performed after obtaining the approval of the Institutional Review Board at Brigham Young University and audio/video consent release was given from all participants. One person in the dyad was randomly chosen to take on the role of the leader and the other participant as the follower. The leader/follower relationship will have direct applications to pHRI as knowledge of the task or dangers might not be known by the robot or the human. The leader was given knowledge of the task and the follower was only told to help accomplish the goal of the leader.

**Figure 1:**
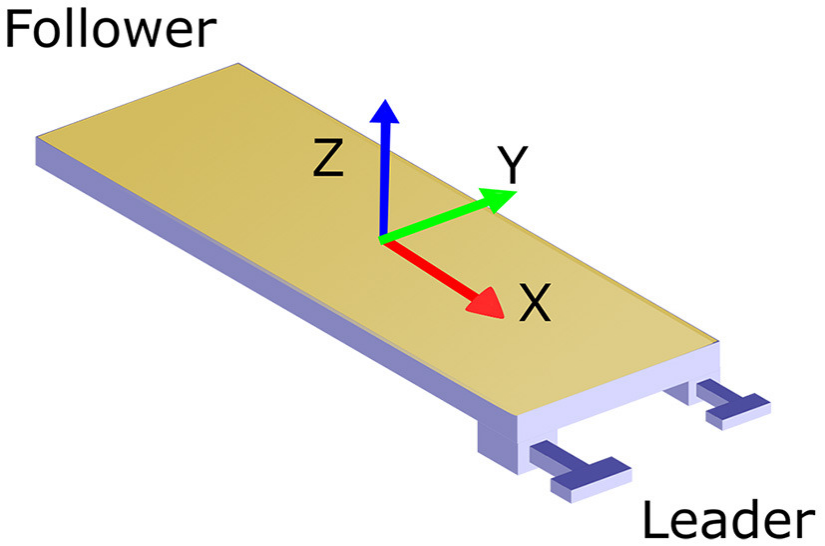
The orientation of the board with red being the X or anterior direction, green being the Y or lateral direction, and blue representing the Z or superior direction. The leader takes the side with the blue handles and the follower takes the opposite side with no handles. The force/torque sensors are attached between the handles and the table.

**Figure 2:**
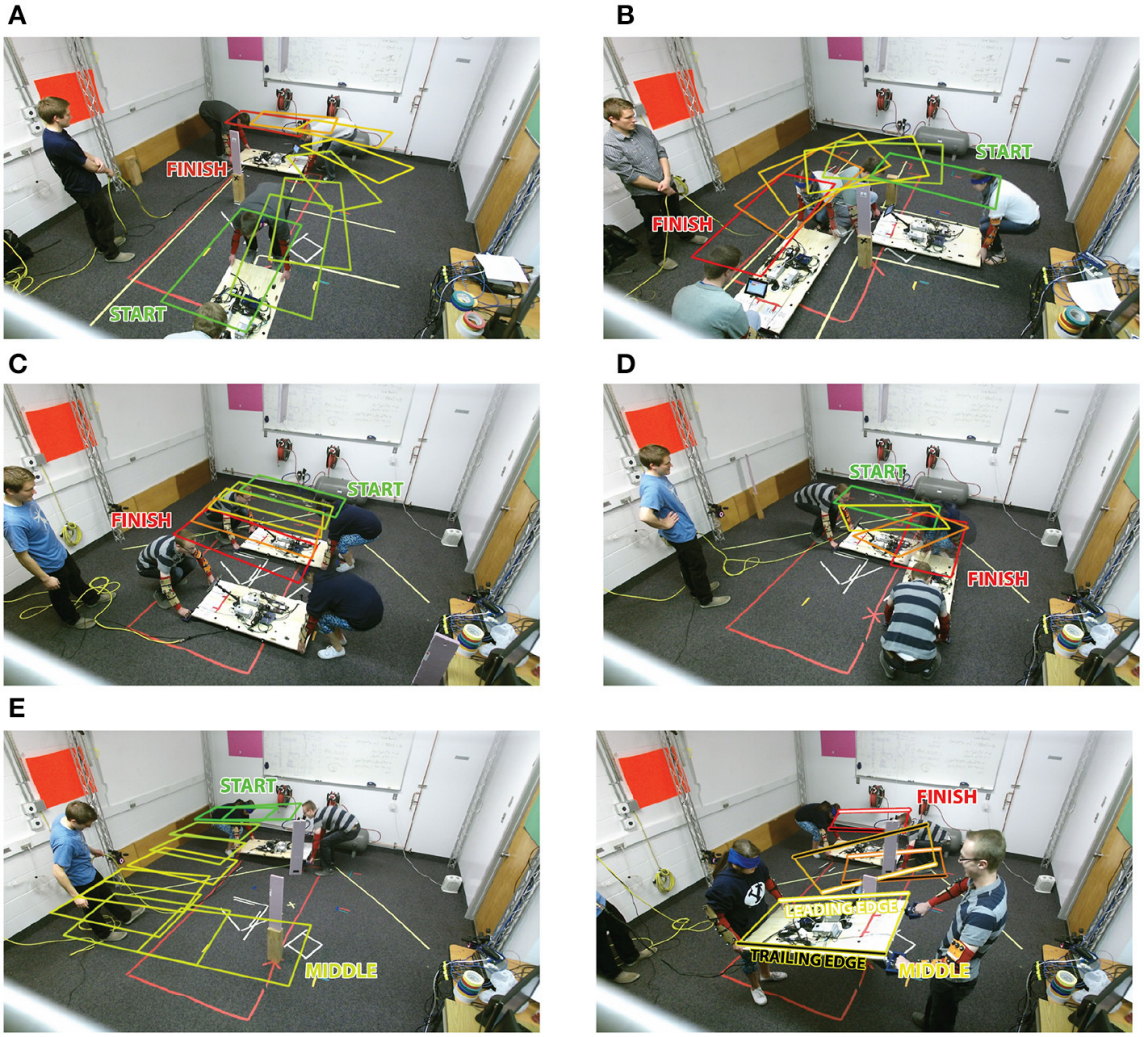
A time-lapse of each task in the study. Colored boxes represent the position of the board at each time step with the colors ranging from green for start to red for the finishing position. The dyad at the start and end positions are also shown for clarity. The tasks are referred to as follows: **(A)** pick and place task, **(B)** rotation and translation, **(C)** pure translation, **(D)** pure rotation, and **(E)** the 3D complex task. The 3D complex task is split into two panels for clarity. The first half of the 3D Complex task required the dyad to walk across the room. The second part of the task required them to lift the board up and over the first obstacle, lower the board underneath the second obstacle and return to the starting position. The second half of the 3D complex task has the trailing and leading edge labeled and colored black and white for clarity.

The object was oriented as shown in Figure 1 with the X, Y, and Z axes matching the anterior, lateral, and superior directions, respectively. Figure 2 describes each of the tasks. The pure translation and rotation tasks, seen in Figures 2C,D, were the most basic of the tasks and only required a translation in the lateral direction and rotation about the superior direction, respectively. The rotation and translation task, seen in Figure 2B, required the team to turn a corner as if in a tight hallway. This task was split into two separate tasks, one with the leader facing forward and one backward. The direction of all the tasks was randomized so that half of the time they translated/rotated left and half of the time they translated/rotated right. The pick and place task, shown in Figure 2A, was similar to the rotation and translation task, but more emphasis was put on the placement of the object and there was more room for them to turn the corner. Lastly, the 3D complex task, seen in Figure 2E, forced the participants to walk in a rectangular shape while avoiding two obstacles. One hanging from the ceiling, the other placed on the floor. This required the dyad to raise and lower the object while angling the object about the anterior direction.

To find the importance of haptic communication compared to other channels, the dyad performed half of the trials without visual and auditory communication. For these restricted communication trials, the follower was blindfolded and the dyad was told that no verbal communication was allowed for the duration of the trial. The motion and forces applied to the object were recorded and analyzed in post-processing. After the set of trials was complete, the dyad was given a brief survey to qualitatively define their experience. The survey contained a list of statements which they were asked to rate on a 5-point Likert scale (Johnson and Morgan, 2016) according to how much they agreed with the statement. The statements and distributions of the answers can be found in Table 1.

**Table 1:** Participants were asked to rate the applicability of each statement from 1-Strongly Disagree to 5-Strongly Agree for the entire duration of all 36 trials.

**Questions**	**Avg**.	**Std**.
My partner was helpful in accomplishing the defined task	4.6	0.5
My partner helped me do the task quickly	4.5	0.6
My partner went slower than I wanted to	2.0	0.9
I felt there was confusion between my partner and me while moving the object	2.2	1.0
I trusted my partner to do the task correctly	4.6	0.6
I felt safe completing the task	4.8	0.4
I trusted my partner to move at appropriate speeds	4.5	0.6
My partner did not push or pull too hard	4.6	0.6
My partner moved in a predictable way	4.3	0.8
I felt like I could complete the task as effectively when they were blindfolded	4.0	0.9
My partner helped me do the task better than I could by myself	4.3	0.8
My partner equally shared the task	4.2	1.0
I consider myself to be assertive	3.7	0.9

*The second column shows the average for all 42 participants and the third column shows the standard deviation of the answers*.

### 2.2. Experiment Protocol and Participants

Our experiment tested 21 human dyad groups. In total, our study consisted of 26 men and 16 women with ages between 18 and 38 with an average age of 22. The majority of the participants were right-handed (38 to 4). Each person signed up for a 1-hour time slot with another participant. The 1-hour time frame allowed for the dyad to complete six different tasks three times blindfolded with no verbal communication, referred to as haptic-only communication (HC), and three times with no restriction on verbal or visual communication, referred to as unrestricted communication (UC). This resulted in 36 total trials which were randomly arranged so the probability of beginning with any task combined with the probability of UC or HC was equal, being 1/12. One individual from each pair was randomly selected as the leader, by a coin flip, and the other was designated as the follower for the duration of all 36 trials. The leader was assigned to hold the side of the object with the handles and was given instructions on how to complete each task through a tablet mounted on the object. The tablet showed both starting and ending positions of the object and was color-coordinated with lines on the floor and posters on the wall to help orient the leader. The follower was not given any instructions on how to accomplish the task although it should be noted that some participants reported knowledge of the goals based on previous repetitions, the obstacles, and lines on the floor.

### 2.3. Materials

A 59 × 122 × 2 cm wooden board was used to simulate an object of considerable length and mass for the experiment. The board was equipped with two ATI Mini45 force/torque (FT) sensors that were placed between the board and two 3D printed ABS plastic handles. The sensors were attached to ATI NET Force/Torque (FT) Boxes which sent the data to the computer at a rate of 100 Hz. In total, the wooden board, and components had a mass of 10.3 kg. The position data was recorded with Cortex Motion Capture software with a Motion Analysis Kestrel Digital Realtime System. Eight Kestral cameras tracked eight infrared markers on the board and eight infrared markers on each participant's arms at 200 Hz. Although participant arm data was not investigated in this paper, it may prove valuable in further studies. The Robot Operating System (ROS) was used to aggregate the data so that it was synced, and time-stamped. In post-processing, the force/torque data was interpolated between each time step because of the time sampling difference. This resulted in access to all of the synced data at a rate of 200 Hz.

### 2.4. Post-processing Algorithms

#### 2.4.1. Motion Data

After all of the experiments were completed the position data of the center of the board was numerically differentiated to obtain velocity and filtered with a low-pass FFT filter at 10 Hz to remove excessive noise due to differentiation. The velocity was then numerically differentiated to calculate acceleration which was also low-pass filtered at 10 Hz.

#### 2.4.2. Interaction Force and Torque

**Figure 3:**
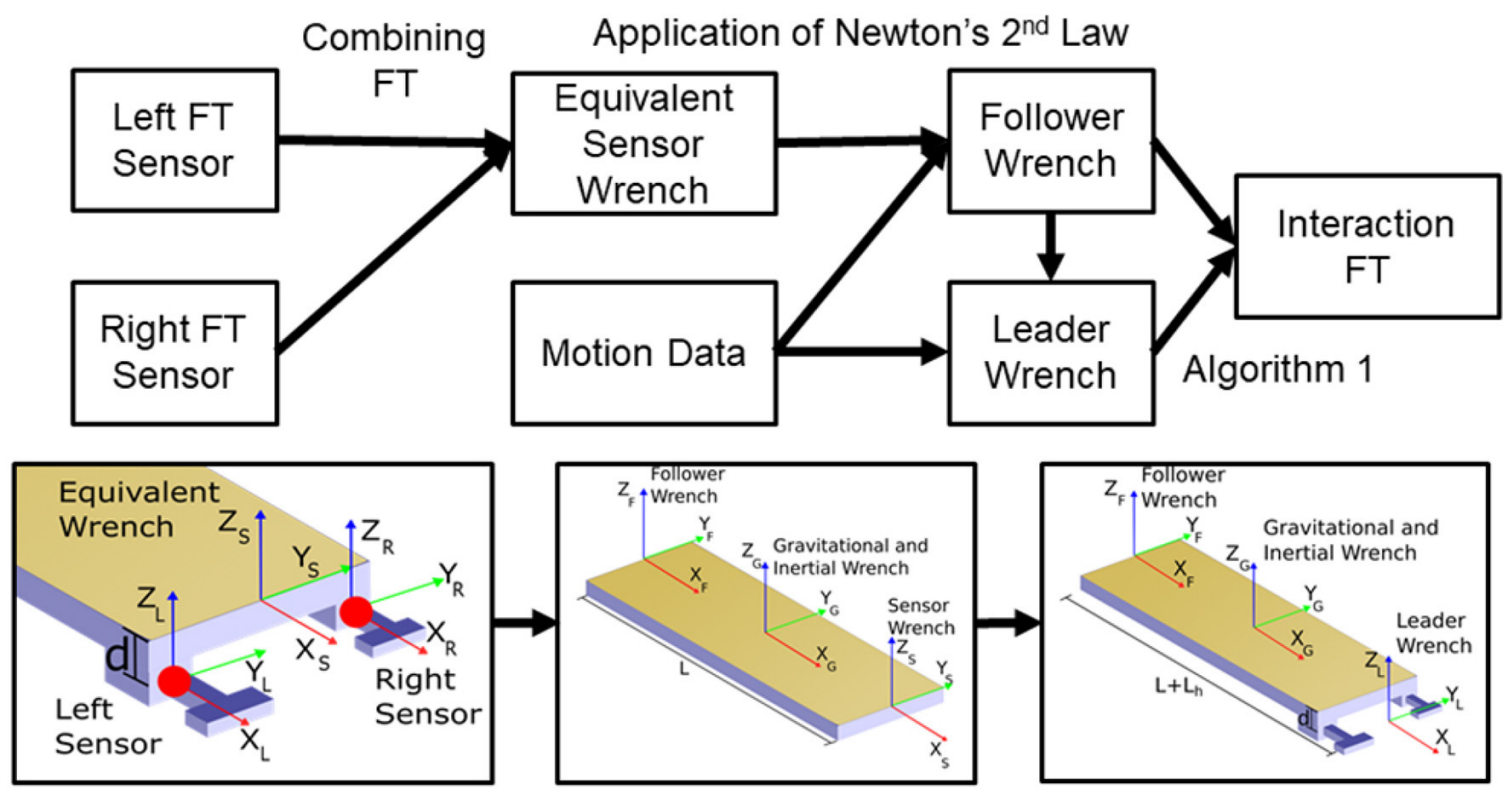
The process of transforming the raw force/torque (FT) data from each sensor into the individual leader and follower FT. The top of the figure explains the process of obtaining the data and the bottom row represents a free-body diagram of the board for each set of calculations. The raw FT sensor data was taken from both the FT sensors (the right and left sides of the leader's end of the board) and was represented equivalently as a single equivalent wrench called the equivalent sensor wrench. The equivalent sensor wrench combined with the inertia and known acceleration of the board allowed for the solution of the individual forces and torques of the follower and leader. This calculation was only valid while the board does not experience any external contact forces (i.e., the calculations were invalid when the board was touching the floor, so the data was cropped to only include data where the board was completely lifted off of the floor).

A wrench is defined as a column vector ℝ^6^ (6 × 1) of forces applied at a point “P” and the torque acting about that point (e.g., ${\left[f_{x,p},f_{y,p},f_{z,p},\tau_{x,p},\tau_{y,p},\tau_{z,p}\right]}^{T}$). The wrench at each point of interest was calculated either directly from the FT sensor data or from the FT sensor data *and* the motion of the board. Since the technique for obtaining these wrenches from a combination of one set of FT sensors and motion data has not been discussed in HHI or pHRI literature (to the authors' knowledge) a detailed description is provided herein. In order to determine the wrench at the follower end of the board, we used a free body diagram to isolate the table from the handles. The FT sensors were placed between the board and the handles which allowed us to know the exact wrench between the handles and the board. The wrench at this point was calculated by combining the FT sensor data from both sensors to obtain an equivalent wrench in the middle of the lateral direction as shown in the leftmost bottom panel in Figure 3. The dynamic analysis was then performed by drawing the wrenches as shown in the middle and right bottom panels in Figure 3. Inertial terms were found from the application of Newton's Second Law (Ginsberg, 2008a). The follower, inertia, and sensor each applied a wrench to the system. The inertial wrench was combined with the gravitational force to represent all of the forces and torques acting on the board. Summing the forces in each direction and taking the moment about the center of the board resulted in six equations with one unknown in each equation. The summation of forces in the X-direction (Equation 1) is shown and was the same for both the Y and Z-directions. Because the wrenches were coincident in the X-direction, the moment equation about the X-direction resulted in the inertial torque equaling the summation of the follower and sensor torques. Equation (2) shows the summation of the torques about the Y-direction and was similar about the Z-direction.

A dynamic analysis of forces in the X-direction gives the following

(1) $$\begin{array}{r} F_{x,s}+F_{x,f}+F_{x,g}=ma_{x} \end{array}$$

where *F* represents force, *m* denotes mass, *a* denotes acceleration, the first letter in the subscript (*x*,*y*,*z*) denotes the direction of the variable and the second letter (*s, f, l, g, i*) in the subscript corresponds to the source of the wrench where the source can be the sensor, follower, leader, gravitational, or inertial wrench, respectively. Summing torques about the Y-direction at the center of mass resulted in

(2) $$\begin{array}{r} \tau_{y,s}+\tau_{y,f}+\frac{L}{2}\left(F_{z,f}\right)-\frac{L}{2}F_{z,s}=\tau_{y,i} \end{array}$$

where τ denotes torque and *L* denotes the length of the board and the torque due to inertia was calculated by Euler's equations of motion (Ginsberg, 2008b) where

(3) $$\begin{array}{r} \tau_{y,i}=I_{yy}\alpha_{y}-\left(I_{zz}-I_{xx}\right)\omega_{x}\omega_{z} \end{array}$$

where α denotes angular acceleration, ω denotes angular velocity, and *I*_*xx*_, *I*_*yy*_, and *I*_*zz*_ are the principle components of inertia.

The same process was implemented to find the leader wrench but by using the length of the board plus the handles for the solution. The wrenches acting on the rigid body were characterized by applied wrenches by the follower, inertia, and leader. The gravitational forces were lumped in with the inertial wrench as shown in Figure 3. The weight of the handles was assumed to be negligible when compared to the board and components therefore the center of mass and total mass remained unchanged. Just like the previous derivation, summation of the forces, and torques in each direction about the center of the board resulted in six equations with one unknown each. The summation of forces in the X-direction is provided in Equation (4) and extends to both the Y and Z-directions. As the handles introduced a slight offset in the Z-direction of the leader wrench, the moment equations are provided for each direction. The X-direction equation was calculated as

(4) $$\begin{array}{r} F_{x,f}+F_{x,g}+F_{x,l}=ma_{x} \end{array}$$

Summing the torques about the X-direction at the center of mass resulted in

(5) $$\begin{array}{r} \tau_{x,f}+\tau_{x,l}+dF_{y,l}=\tau_{x,i} \end{array}$$

where *d* denotes the length from the board to the handles in the Z-direction. Summing the torques about the Y-direction at the center of mass resulted in

(6) $$\begin{array}{r} \begin{array}{c} \tau_{y,f}+\tau_{y,l}-dF_{x,l}-\left(\frac{L}{2}+L_{h}\right)F_{z,l}+\left(\frac{L}{2}\right)F_{z,f}=\tau_{y,i} \end{array} \end{array}$$

where *L*_*h*_ denotes the length of the handles. Summing the torques about the Z-direction at the center of mass resulted in

(7) $$\begin{array}{r} \tau_{z,f}+\tau_{z,l}+\left(\frac{L}{2}+L_{h}\right)\left(F_{y,l}\right)-\left(\frac{L}{2}\right)F_{y,f}=\tau_{z,i} \end{array}$$

Using this method we found the complete wrenches for the leader and follower. The process for obtaining the interaction FT data is shown in Figure 3.

As several researchers have pointed out the importance of interaction forces (forces internal to the dyad; i.e., forces not contributing to the motion of the object), these were also calculated and analyzed. The definition of interaction forces was based on Groten et al. (2009) (see Algorithm 1 lines 13–17) and was extended to include interaction torque and gravitational effects by using Algorithm 1. The algorithm takes in the follower and leader force and torque (FT) and checks for gravitational effects. Since gravity can make the definition of interaction FT ambiguous, this algorithm essentially subtracts the effect of the gravitational force from the force with the opposite sign. The algorithm then takes the smaller of the leader or follower FT magnitudes, as long as they are opposite signs. If the two FT have the same sign then the interaction force is set to zero. As an example of the algorithm, consider the possibility that the board is slightly tilted about the Y axis i.e., the board is slanted downwards toward the follower. This means that in the coordinate frame of the FT sensors there is a small force due to gravity in the negative X direction. Let us say that the X direction forces are as follows: follower force is −5 N, the leader force is 10 N, and the force of gravity is −1 N. Because we are dealing with forces, lines 4–12 in Algorithm 1 apply. Because the signs of the follower and leader forces do not match (line 5) and the sign of the follower force and gravity does match (line 6), the force of gravity is added to the leader force, thus the force of the leader is now 9 N. Because the leader and follower forces have opposite signs, there exists an interaction force and its magnitude is set at 5 N.

**Algorithm 1:** Interaction Force/Torque.

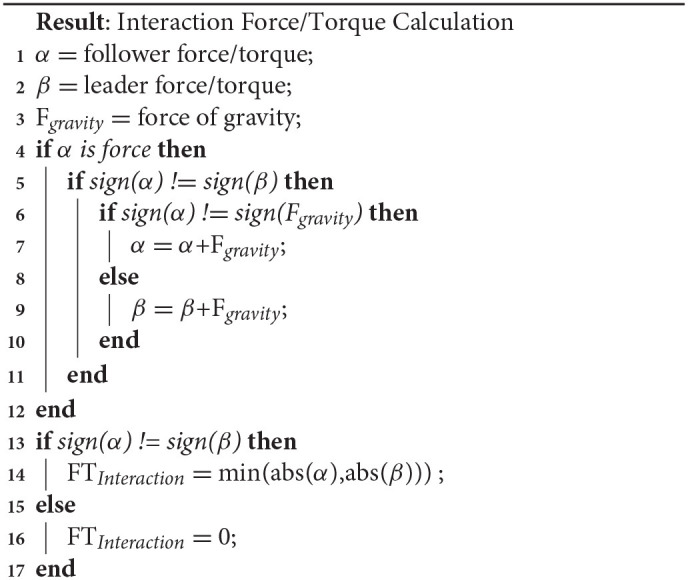

To simplify the analysis, the magnitude for each of the metrics was calculated using the two-norm. For example, assume that at a single timestep the interaction force is 3, 4, and 5 N, respectively for the X, Y, and Z-directions, this would be simplified to 7.07 N. The median and 95th percentile values of each trial were compared to find trends in the data. The 95th percentile value was used instead of the maximum value to remove any uncharacteristic spikes in the data.

For the interaction force and torque (FT) specifically, the 95th percentile value was also preferred over the median value because we hypothesize that the leader most likely employs smaller bursts of large forces to communicate. This was confirmed by the data because the 95th percentile of interaction FT was usually more correlated with the other metrics than median values except where noted in our results. For velocity, only the median values were included in the analysis as numerical integration can be very susceptible to noise and median values had stronger correlations to completion time specifically. The raw completion times were retained and used in subsequent analyses without any summary statistics.

## 3. Results

Overall, we investigated four different aspects of human-human dyad co-manipulation. In the first section, the overall performance and group dynamics were considered. The term group dynamics denotes that there were some dyads who performed in a significantly different manner from other dyads in terms of the metrics we used to quantify performance. Secondly, we considered interaction force and torque (FT) and their correlation to other metrics described in section 3.1. Thirdly, spectral analysis revealed that there were two peak frequency ranges which humans tend to exhibit while carrying an object. Finally, in the fourth section, the effects of restricting communication to haptic-only feedback is explored.

### 3.1. Performance and Group Dynamics

The performance metrics selected were based on what can be measured by a robot and are commonly implemented for control in pHRI. We compared interaction force and torque (FT), linear and angular velocity, repetitions, and completion time. While we recognize that this is far from a complete set of all relevant metrics, we maintain that these metrics will allow us to develop novel legible and predictable controllers as well as compare current controllers established in the literature to actual human-human performance. The distributions of these metrics represent nominal human-human dyad performance. This is classified as nominal performance because the participants were at least age 18, so they have most likely had several experiences moving an object with another human, and no qualitative instructions that would change their nominal behavior, such as “move quickly,” were given to the dyads before the trials.

Table 2 shows our findings for the distributions of motion and haptic feedback. To better understand the interaction force and torque (FT) distribution, the data points with no haptic interaction, i.e., interaction FT equal to zero (see Algorithm 1) were excluded from the distribution for FT. The last column in Table 2 shows how often there was an interaction force/torque between the dyad as a percentage of time. As can be seen, there were haptic interactions applied more than 70% of the time excluding interaction torque in the Y-direction. Due to the high percentage of time that haptic interaction was occurring, haptic interaction should not always be minimized in a pHRI controller as would be the case if the controller were designed to minimize wasted energy. The relatively low values for interaction torque in Y can most likely be linked to the fact that the leader was holding cylindrical handles which would make it difficult for the leader to apply a torque about the Y-direction. The Y-direction interaction torque distribution is still potentially valuable data because pHRI controllers should know the distribution of interaction torques to expect/apply when the human is holding cylindrical handles. Because of the way in which interaction force was calculated, interaction force in the Z-direction rarely occurred as the board was usually oriented normal to the superior direction.

**Table 2:** Distribution of Interaction FT and velocity data for all 36 trials of all 19 groups.

**Data**	**Median**	**Q1**	**Q3**	**Max**	**% of time interaction occurred**
Linear velocity in X (m/s)	0.12	0.04	0.28	1.10	N/A
Linear velocity in Y (m/s)	0.11	0.04	0.29	1.39	N/A
Linear velocity in Z (m/s)	0.04	0.02	0.11	1.46	N/A
Angular velocity in X (rad/s)	0.07	0.03	0.13	2.48	N/A
Angular velocity in Y (rad/s)	0.05	0.02	0.10	1.82	N/A
Angular velocity in Z (rad/s)	0.10	0.04	0.25	3.63	N/A
Interaction force in X (N)	8.38	3.92	14.00	64.64	76.09
Interaction force in Y (N)	2.90	1.36	5.24	38.39	70.58
Interaction torque in X (Nm)	2.74	1.26	4.86	28.74	94.26
Interaction torque in Y (Nm)	0.17	0.07	0.32	7.29	38.10
Interaction torque in Z (Nm)	2.89	1.31	5.53	34.63	81.08

*All values of zero are removed for the interaction FT to better understand the distribution when interactions are happening. The last column shows the percent of time that interaction FT were being applied. Interaction Torque in the Y-direction may have been limited because of the design of the handles*.

**Figure 4:**
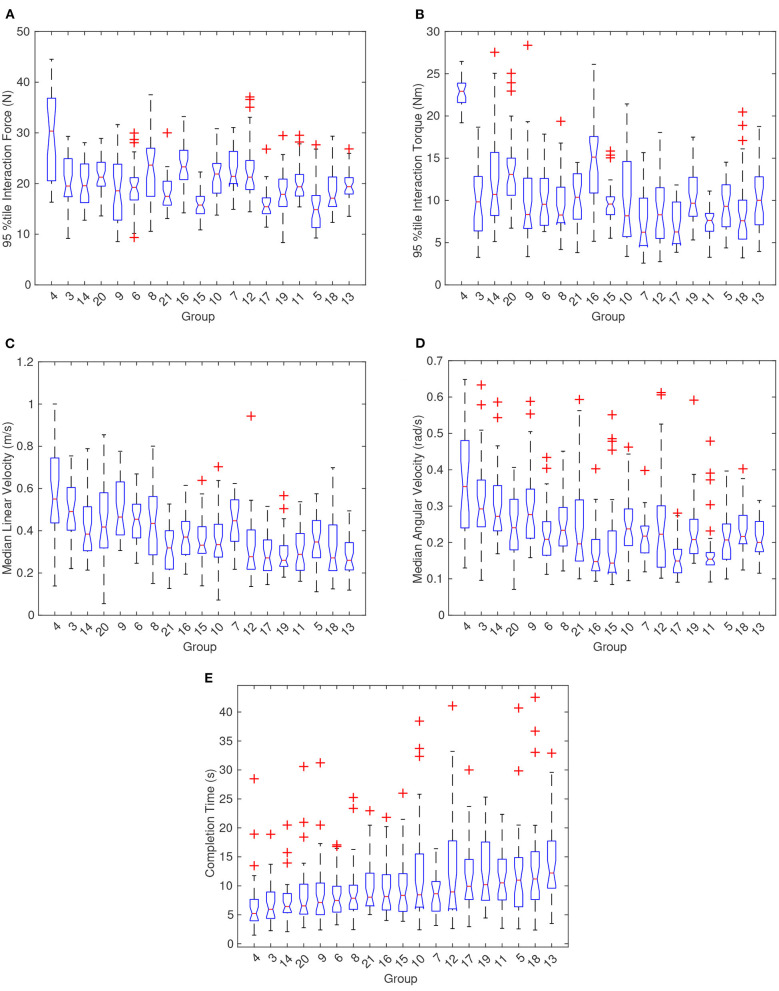
Boxplots showing the distribution of the proposed metrics according to each group. No data exists for groups 1 and 2. **(A,B)** Depict the 95th percentile interaction force and torque (FT), respectively, between the dyads. **(C,D)** Show the distribution of median linear and angular velocity for each dyad. **(E)** Illustrates the difference in completion times for all of the dyads. The groups were sorted by increasing completion times and so it should be noted that the trend for median linear and angular velocity were opposite of the trend for completion time.

In terms of group dynamics, we found that some groups varied significantly from each other in terms of completion time, linear and angular velocity, and interaction FT, as shown in Figure 4. Figure 4 has a subfigure for each metric with 19 boxplots that show the distribution of the stated metric for each dyad. Note that the boxplots show the distribution of the selected statistic and not the entire distribution of all data. The red plus symbols represent outliers determined by data points >1.5 times the interquartile range plus the third quartile or <−1.5 times the interquartile range plus the first quartile. The notches in the boxplots (i.e., areas where the box plot narrows to a minimum width) represent the 95% confidence interval for the median of the statistic of the metric for each dyad. Thus, as can be seen from Figure 4, there were several groups for which we rejected the null hypothesis for the same median at the 0.05 significance level. For example, using a two-sample *t*-test for equal means on completion time data, the extreme groups of 4 and 13 had a *p*-value of 0.00005. The p-value for the same test using groups 15 and 16 reported a value of 0.9744 which means that we cannot assume that groups 15 and 16 would have different mean completion times if allowed to continue performing trials. The null hypothesis (that the groups have equal means) was also rejected for the rest of the metrics for groups 4 and 13, except for interaction forces which were quite similar. However, multiple other groups could not be assumed to have the same overall mean interaction forces, for example see groups 19 and 12. This shows that although some dyads can be similar, some dyads will most likely perform significantly different, and thus an adaptive element or a training period will be required in pHRI controllers.

**Figure 5:**
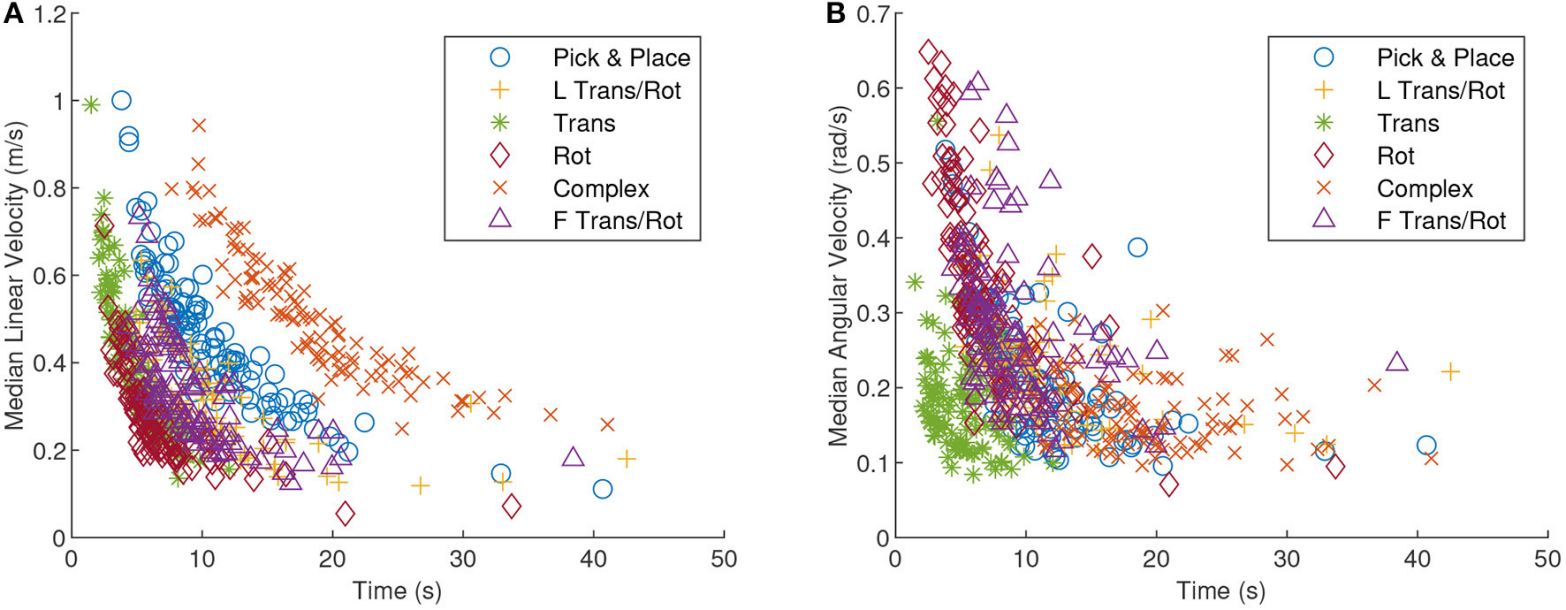
A scatter plot of the median linear velocity **(A)** and median angular velocity **(B)**, as a function of completion time. The tasks are all delineated by different shapes and colors. A strong correlation between linear velocity and completion time should be noted.

The groups within each graph in Figure 4 were ordered by increasing median completion time. An interesting note is that 95th percentile interaction force and torque are not strongly correlated with completion time although an inverse correlation between completion time and velocity can be observed. The Kendall's Tau correlation value between median linear velocity and completion time averaged to −0.66 across all tasks which suggests that median linear velocity and completion time had a strong negative correlation. Replacing linear velocity with angular velocity yielded a Kendall's Tau mean of −0.42 across all tasks. Looking at the relationship more closely in Figure 5, completion time and linear velocity were strongly correlated, but the relationship was not linear in nature. A Pareto frontier can be seen in Figure 5 and thus an optimal median speed for a given task could be selected based on this curve and the desired cost function. In other words, trying to always move faster will give diminishing results in terms of completion times. The same can be said for angular velocity although the correlation was not as strong.

Robots co-manipulating with a human could pick velocities from a range of values on this curve. The robot will also be able to weigh the cost of the expended work to move at a certain velocity given the urgency of completing the task in a timely manner. A robot designer will also want to know that angular velocity for some tasks is not necessarily strongly correlated with completion time. For the pure translation task specifically, completion time was relatively weakly correlated with angular velocity (Kendall's Tau of −0.22 for translation task vs. −0.62 for rotation task) and thus the best thing for the robot to do may be to slow down to correct for rotation due to the angular motion and then speed up in the linear direction.

**Figure 6:**
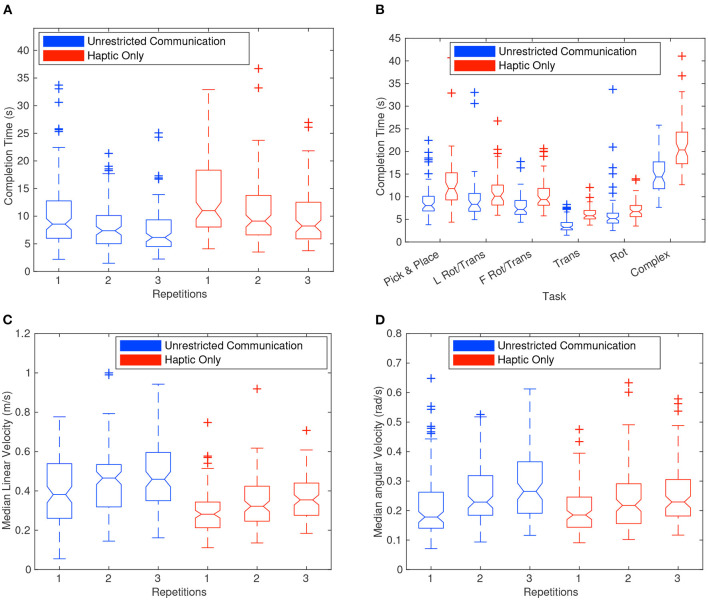
Boxplots showing the distribution of completion times divided by repetition **(A,B)**. It should be noted that the haptic-only communication (HC) complex task was significantly longer in completion time than the other tasks. The translation task with unrestricted communication (UC) also was significantly shorter than the other tasks. **(C,D)** Show boxplots of the distribution of median linear and angular velocity for each repetition. In **(A,C,D)**, a significant amount of learning can be perceived between the dyad in terms of the reduction in completion time and the increase in speed.

Figure 6A shows the correlation between the number of repetitions and the completion time. The six boxplots represent the distribution of all completion times for each repetition and are also divided out by haptic-only (HC) and unrestricted communication (UC). Figures 6C,D are very similar but show the distribution for median linear and angular velocity instead of completion time. As seen in Figure 6A, the completion time dropped significantly as the number of repetitions increased for both HC and UC. The overall median of the completion times for each repetition dropped from 9.9 to 7.9 s and lastly to 7.0 s. This curve appears to quickly approach a constant value as the number of repetitions increases. Indeed a *t*-test for equal means when comparing repetitions at the 0.05 significance level rejected the null hypothesis for completion time between repetitions 1 and 2 but failed to reject the null hypothesis between repetitions 2 and 3 (*p*-values were 0.00004 and 0.0531, respectively). It should be noted that the decrease in completion times was matched with an increase in velocity. The drop in completion times and the increase in velocity was most likely a function of the dyad becoming familiar with each other and the tasks. Videos of a dyad showing learning for the pure translation task can be found here: Rep 1: https://youtu.be/Zt47CZNcDuU, Rep 2: https://youtu.be/Q_vOwpJM27A, and Rep 3: https://youtu.be/5KlqhBIyOg8. On each repetition, there was less confusion in the dyad as inferred in the amount of time it took to complete the task and in the amount of deviation from a static orientation.

Most of the very wide distribution of completion times for each group can be explained by the difficulty of the tasks themselves, as shown in Figure 6B. This figure shows 12 boxplots of the distribution of completion times, one for each task required in the experiment, and further divided by haptic-only (HC) and unrestricted (UC) communication. The complex task involved the longest distance from start to end position and thus claimed the top spot as the longest task with a median completion time of 20.3 s for HC trials. At the other end of the spectrum, the translation task was the quickest with a median time of 3.2 s for the UC trials. As discussed later, the HC tasks had significantly longer completion times than UC. Also of note was that the distribution of completion times for the rotation and translation task, when the leader went first around the corner vs. when the follower went first, were very similar and failed to reject the null hypothesis of a two-sample *t*-test for equal means with a *p*-value of 0.08 and 0.64 for UC and HC, respectively.

### 3.2. Interaction Force and Torque

As shown in Table 2, interaction forces and torques (FT) occurred during a majority of each task, occurring at least 70% of the time except in the case of torques about the Y-direction and forces in the Z-direction as discussed in section 3.1. Interaction FT are especially interesting because the dyad was exerting extra effort that was not serving to directly facilitate the mechanics of completing the task.

**Figure 7:**
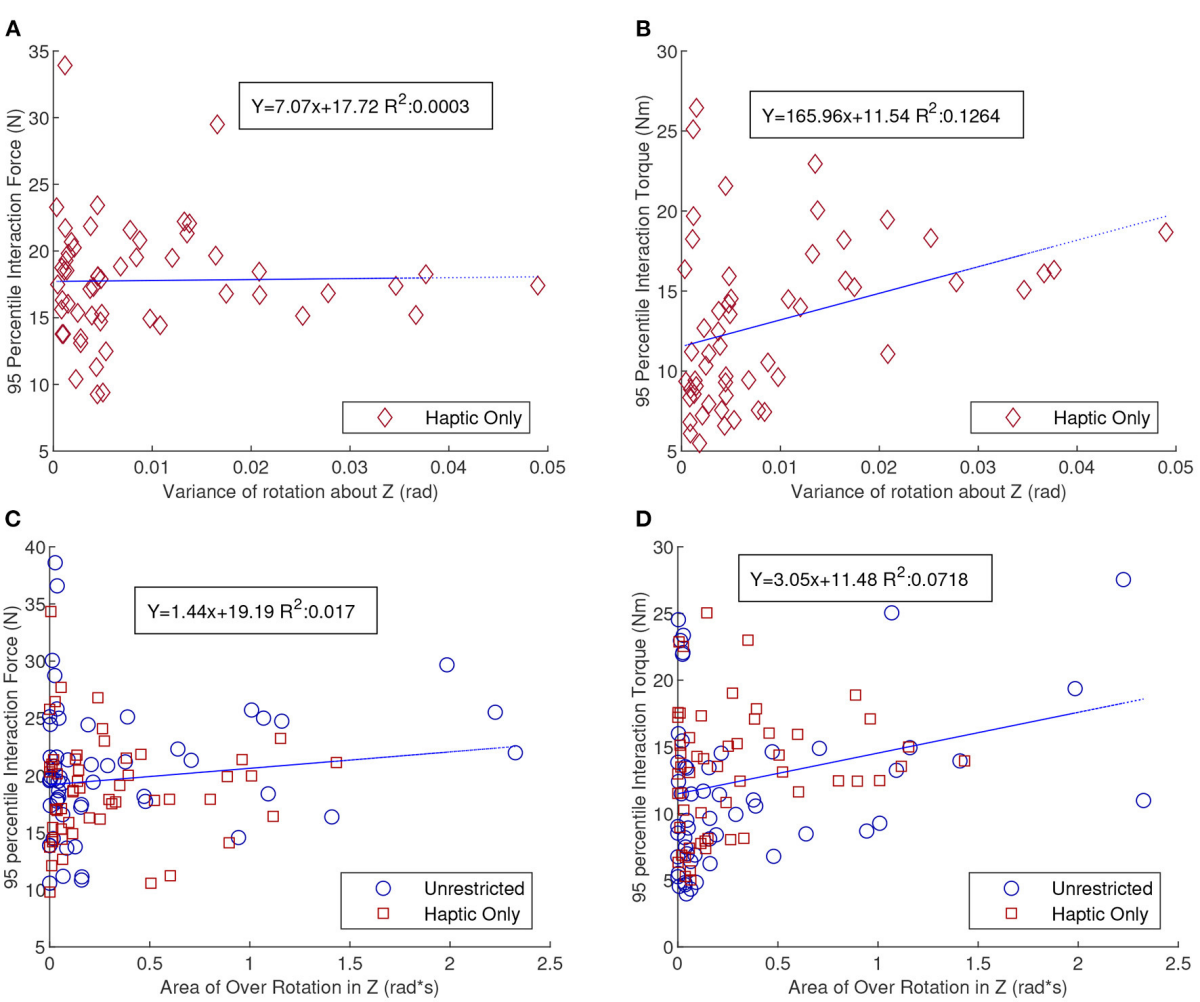
Scatter plots of the 95th percentile interaction force **(A)** and torque **(B)**, as a function of variance of the orientation about the Z-direction. This is only shown for the pure translation HC tasks. **(C,D)** Show scatter plots for the 95th percentile interaction force and torque (FT), respectively, as a function of area of over-rotation of the orientation about the Z-direction. This is shown only for the pure rotation tasks. Of special note in these figures is the positive correlation for interaction torque and the lack of correlation for interaction force with the proposed measures of synchrony.

The pure translation and rotation tasks are particularly useful in trying to understand the interaction force and torque (FT) because of their simplicity. The pure translation task would essentially be perfect in terms of efficiency or wasted effort if the dyad were to keep the board orientation as close to constant as possible. Variations in the orientation of the board, especially about the superior direction, would indicate some amount of disagreement either for the direction, or speed. Thus, we propose that the variance of the orientation about the Z-direction for the pure translation task could be transformed into a measure of synchrony. Figures 7A,B show the total magnitude of the 95th percentile interaction FT as a function of the variance of rotation about the Z-direction, respectively. This behavior is only plotted for the HC pure translation tasks as there was almost no variation for UC tasks. Sight seemed to help the dyad maintain static orientation which confirmed the finding in Parker and Croft (2011). Figure 7A shows that the R-squared value between interaction force and variance of orientation about Z was almost zero, however the R-squared value for the 95th percentile interaction torque, Figure 7B, indicates a positive linear correlation with a R-squared value of 0.1264. Although it is relatively small, it still exceeds a threshold (0.1) suggesting an explanation for some of the given variance found in human-generated data (Falk and Miller, 1992). This would indicate that the 95th percentile interaction torque could indicate disagreement for a pure translation task.

Exploring the pure rotation task, over-rotation of the board may also be a sign of disagreement or at least a lack of synchrony. To get an estimate of the amount of disagreement, we calculated the time integral of overshoot of the final goal orientation. Figures 7C,D show that the pure rotation tasks have similar results as the pure translation tasks but with weaker conclusions. Since both HC and UC have significant over-rotation both are included in the graphs and the blue line of best fit describes both sets of data. Although more studies will be required to absolutely verify these correlations, given the small R-squared values and variance in the data, high interaction torques as opposed to high interaction forces between the dyad seems to communicate that they were not in sync with each other.

**Figure 8:**
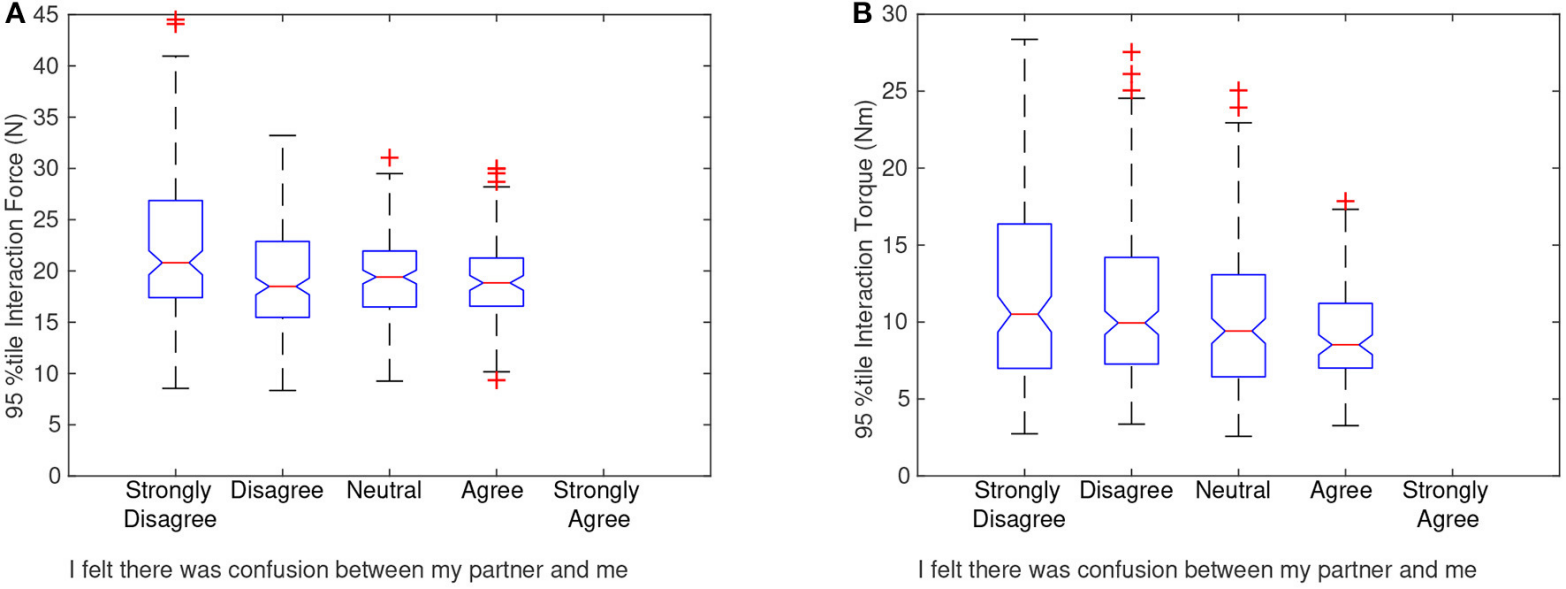
Boxplots showing the distribution of haptic interactions for each possible survey answer. **(A)** Shows the 95th percentile interaction force and **(B)** shows the 95th percentile interaction torque of each trial compared with the post-experiment survey answer of the follower. Because each dyad completed only one survey, the follower rating for that dyad was paired with the 95th percentile interaction force and torque values, giving 36 different points for each dyad. After the 95th percentile values and survey responses were paired, the boxplots were created to better visualize the distribution. The follower was asked to rate the statement “I felt there was confusion between my partner and me while moving the object” using the 5-point Likert scale. As can be noted from the graphs, the followers who strongly disagreed with the statement tended to have trials that were higher in interaction force and torque (FT) in contrast to those who agreed with the statement.

After the dyad was finished with the study they were asked to rate the statement, “I felt there was confusion between my partner and me while moving the object,” using the Likert scale from 1-strongly disagree to 5-strongly agree. Figures 8A,B show the survey response of the followers matched with the 95th percentile interaction force and torque (FT), respectively, of each trial the dyad performed. Boxplots are shown to capture the distribution of group/trials for each survey answer. This was done to preserve the spread of the trials and the extreme values that may have influenced the survey data. The followers' responses were specifically interesting because the followers had no foreknowledge of the end goal and had to infer all of their actions based on the leaders' communication. As seen in Figure 8, the high interaction forces and torques were correlated with less confusion, and a smaller range of high interaction FT was correlated with higher confusion. Based on this correlation, higher interaction FT should be exerted to some extent to reduce confusion. This is consistent with previous research by Sawers et al. (2017), where they found that expert dancing dyads exerted higher interaction forces than novice dyads. While we do not yet understand why the dyads who were less confused had higher forces and torques, one answer could be that high interaction forces and torques help the dyad be more sensitive to the leaders' communication.

**Figure 9:**
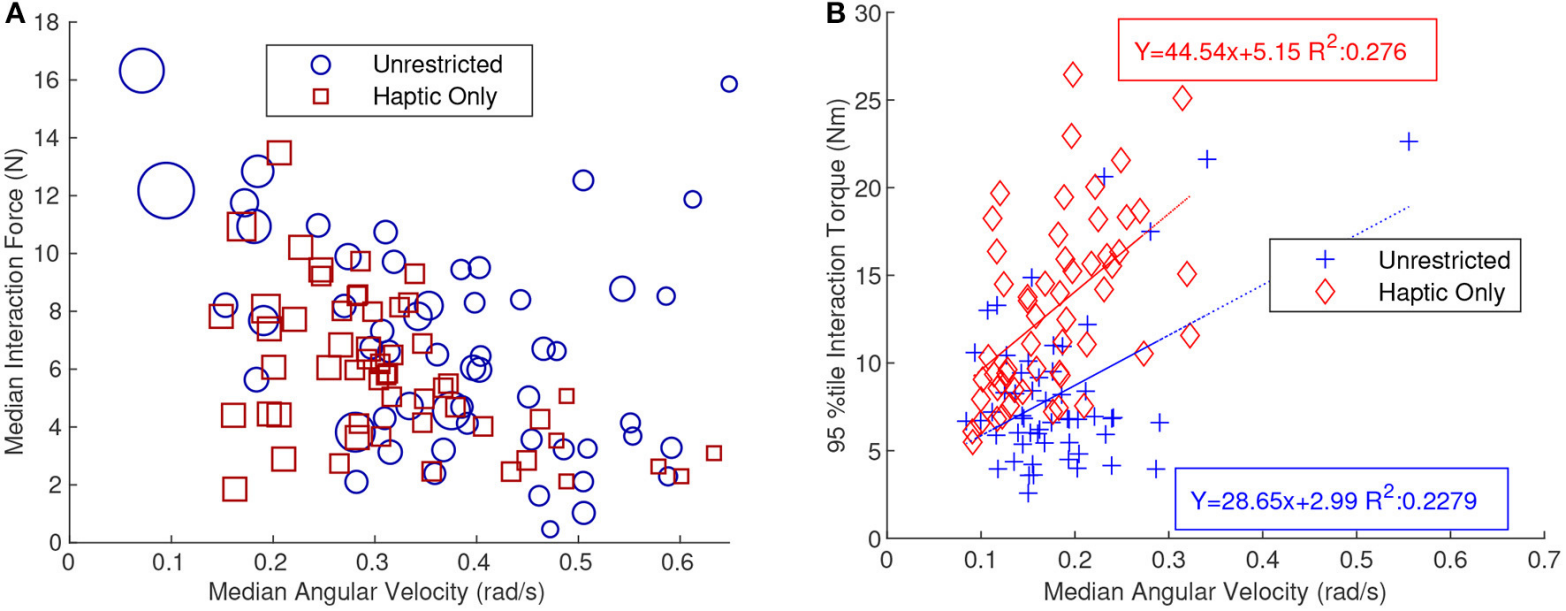
**(A)** Shows a scatter plot of the median interaction force as a function of median angular velocity. This graph shows only trials that were part of the pure rotation tasks. A strong linear negative trend is shown and thus median angular velocity generally increases as the median interaction force decreases for the pure rotation task. The size of the markers was also based on the relative completion time. Therefore, a larger marker will have a longer completion time. **(B)** Shows a scatter plot with the 95th percentile interaction torque as a function of median angular velocity. This graph shows only trials for the pure translation task. The data shows a positive correlation between the two metrics. A line of best fit was also included on the plot for both the haptic-only (HC) and unrestricted (UC) communication tasks. The positive trend was especially interesting since the pure translation task required no angular movement.

Figure 9A shows the median interaction force as a function of median angular velocity for the rotation only tasks. The blue circles represent the UC tasks and the red squares represent the HC tasks. The size of the markers is based on the completion time, the larger a marker the longer the completion time. The graph shows a significant negative correlation with a Kendall's Tau of −0.249 and −0.336 for UC and HC, respectively. This shows that, at least for tasks with rotation about the Z-axis, a smaller interaction force correlated with a faster angular velocity. Figure 5B shows that angular velocity was negatively correlated with completion time and this was confirmed by the size of the markers in Figure 9A diminishing when moving from the top left to the bottom right.

Figure 9B shows the 95th percentile interaction torque as a function of median angular velocity for only the translation tasks. This was interesting because in this task angular velocity was relatively weakly correlated with completion time (Kendall's Tau of −0.22 for translation only vs. −0.62 for rotation only) and angular velocity was not required at all for the task. The data in Figure 9B, received a Kendall's Tau of 0.03 and 0.42 for UC and HC, respectively. The Kendall's Tau correlation value was low for UC because it discounted the higher values which may be considered outliers. By applying a linear regression to the data an R-squared value of 0.23 was found which validates the positive correlation. This trend suggests that higher interaction torques correlate with higher median angular velocities.

### 3.3. Spectral Analysis

**Figure 10:**
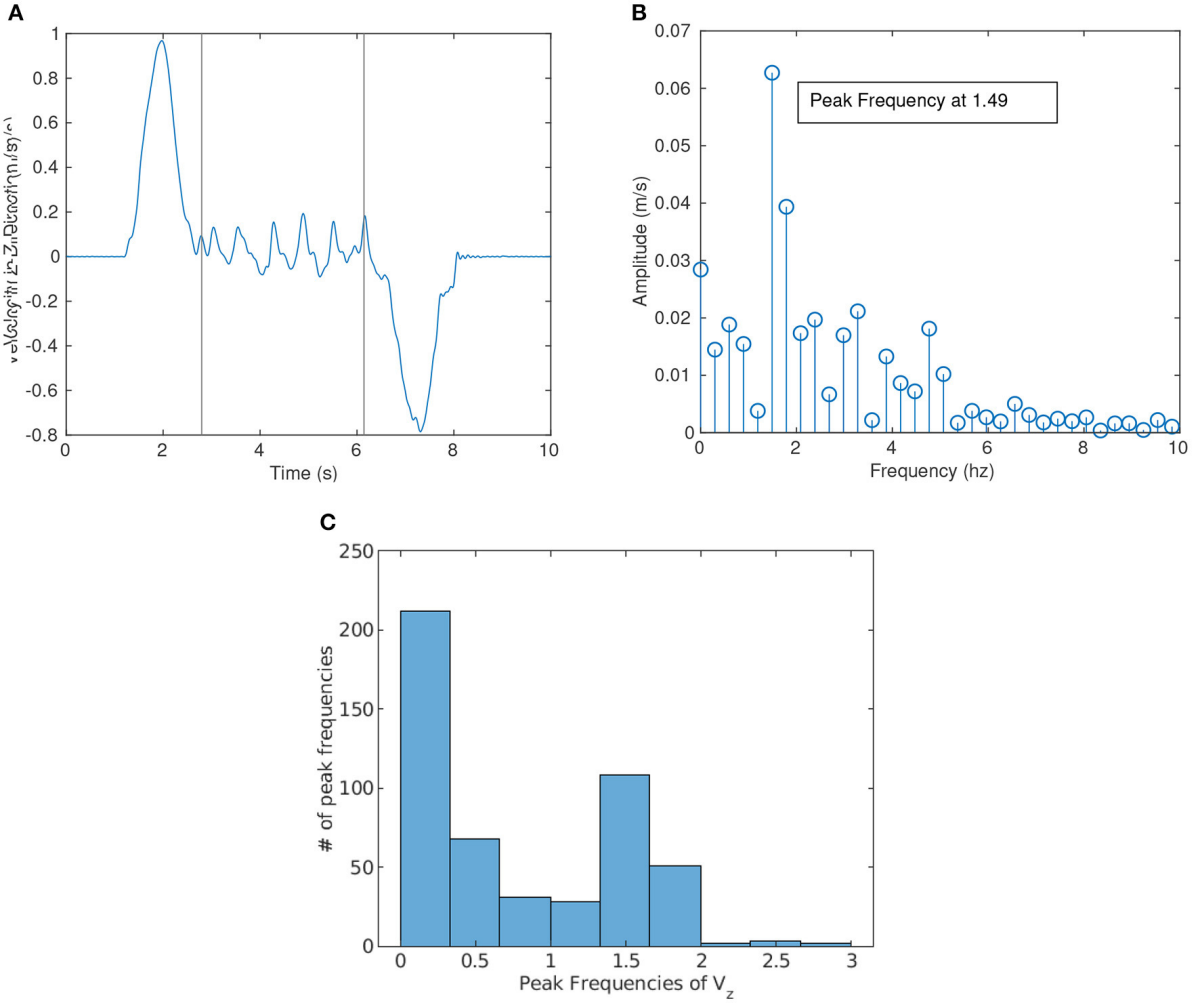
**(A)** shows the velocity in the Z-direction as a function of time for a single trial of the pick and place task and **(B)** shows the Fast Fourier Transform (FFT) of that same signal. A peak frequency of 1.49 can be seen in the graph. **(C)** Shows a histogram of the peak frequencies of velocity in the Z-direction for all trials. Two Peaks can be seen in the graph above: one peak below 0.33 Hz, and the other between 1.33 and 1.66 Hz.

The motion of the board (or object being carried) is of particular interest to robotics researchers since control is usually linked to the motion of the board as an objective. We observed that there was measurable oscillatory motion in the superior direction of the board. Figure 10A shows the velocity in the Z-direction as a function of time from a single trial of the pick and place task and is an example of the oscillatory motion observed. A Fast Fourier Transform (FFT) analysis was performed on the motion of all trials to find if there was a specific frequency at which the board oscillates (essentially up and down). Figure 10B shows a graphical representation of the FFT with the magnitude and frequency of the FFT for the velocity of the object in the Z-direction shown in Figure 10A. Because an FFT filter was applied to remove all noise above 10 Hz, the graphs do not include any data above 10 Hz.

To make sure that the sampling did not introduce problems in the FFT, only samples that were longer than 3 s were included in the spectral analysis. This corresponds to a discretization of at least 0.33 Hz and smaller. To further protect the data, the position and thus velocity data from the complex task was cropped so that the portion of data coming from the dyad lifting the board above and below the two obstacles was removed. This provided a combined set of trials from all tasks in which no motion in the Z-direction was required.

The peak frequency was gathered from each trial to find the most common or natural frequency exhibited by human dyads. As shown in Figure 10B, the peak frequency of this specific trial was 1.49 Hz which was then represented as a single data point for Figure 10C. Figure 10C shows the distribution of peak frequencies for all trials longer than 3 s. The FFT analysis shows two distinct bumps, one from 0 to 0.33 Hz and the second between 1.33 and 1.66 Hz. The first bump most likely corresponded to intentional motion. When considering the lifting of the board, a similar peak around 0.4–0.5 Hz was found. These findings were consistent with Parker and Croft (2011), who found that humans struggled to respond well to MJ movements above 0.5 Hz. The second bump may then correspond to the natural gait of humans and may be a natural part of any human-human interaction.

### 3.4. Haptic-Only vs. Unrestricted Communication

**Figure 11:**
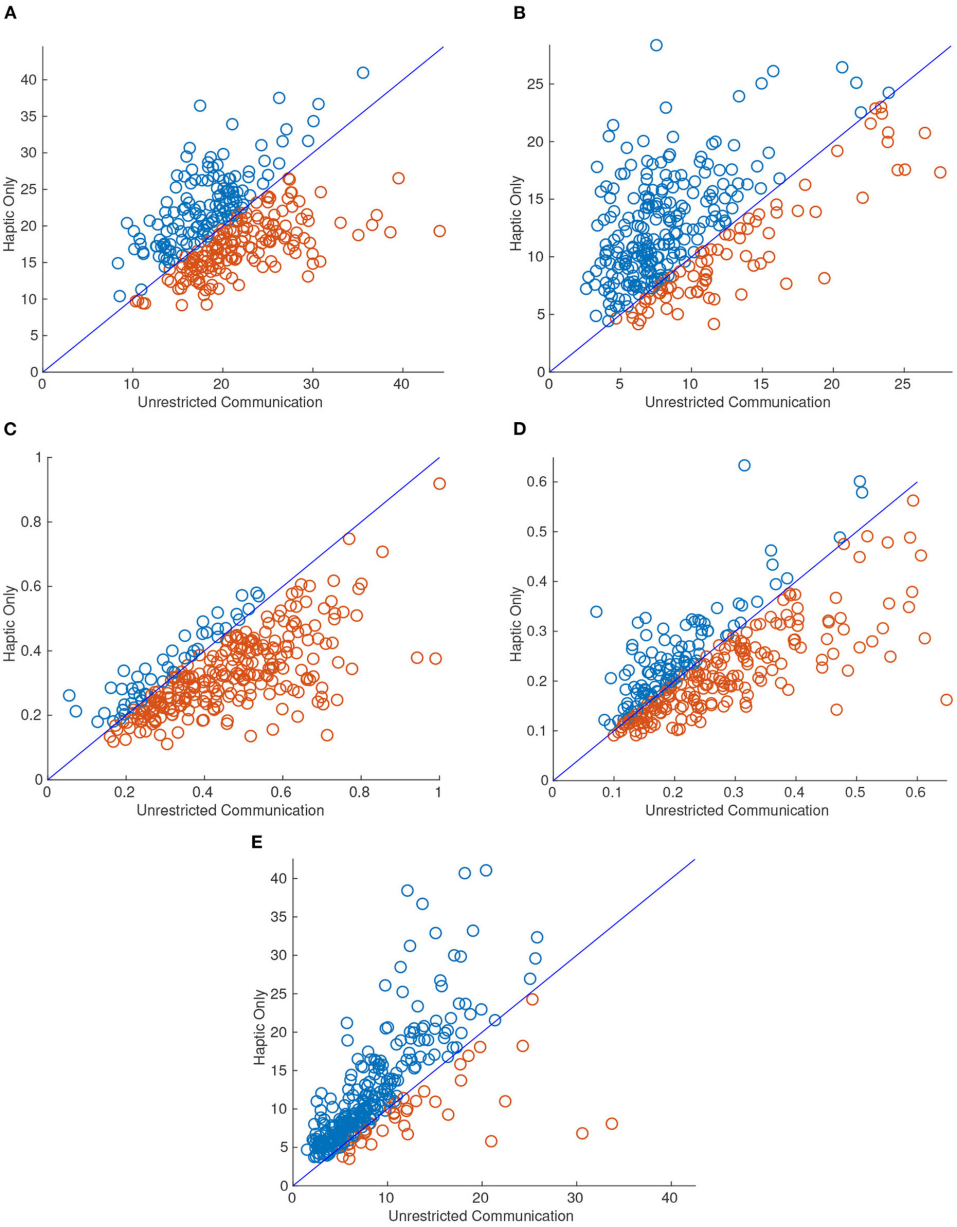
Scatter plots for 95th percentile interaction force **(A)**, 95th percentile interaction torque **(B)**, median linear velocity **(C)**, median angular velocity **(D)**, and completion time **(E)**. The X value of each point corresponds to the metric value when the dyad attempted the task with unrestricted communication (UC) and the Y value corresponds to when the dyad performed the task with haptic-only communication (HC). Thus, there were half as many points as there were trials. Blue and red markers represent an increase and decrease, respectively, in the metric when the dyad performed the same task with HC. All of the metrics had significantly different means from HC to UC at the 0.01 level except for 95th percentile interaction force.

A significant change was observed in most of the performance metrics when the dyad was limited to haptic-only communication (HC). Most notable was completion time which was significantly reduced when only haptic feedback was allowed as seen in Figure 11. Figure 11 shows a scatter plot with the difference between HC and UC in each of the metrics. For each point, the X value was the dyads performance for the task with unrestricted communication (UC) and the Y value was the same task *and* repetition but restricted to haptic-only feedback (HC). The diagonal line represents y = x and therefore any points above the line indicate that the dyad performed higher in that metric when restricted to HC compared with the UC tasks. The markers are colored blue and red for those above and below the diagonal line, respectively, for ease of interpretation.

As can be inferred from Figure 11, the 95th percentile interaction force was the only metric that did not reject the null hypothesis for a two-sampled *t*-test between the two communication modes for equal means with a significance level of 0.01. The *p*-value for the 95th percentile interaction force was 0.39. However, on average, dyads exerted a higher 95th percentile interaction force and torque (FT) on each other when restricted to HC. Not surprisingly, the average median linear and angular velocity was lower when restricted to HC. It also follows that dyads also took a longer time to complete each task when limited to HC. In fact, the completion time increased by 47%, on average, when the dyad completed the same task with HC.

**Figure 12:**
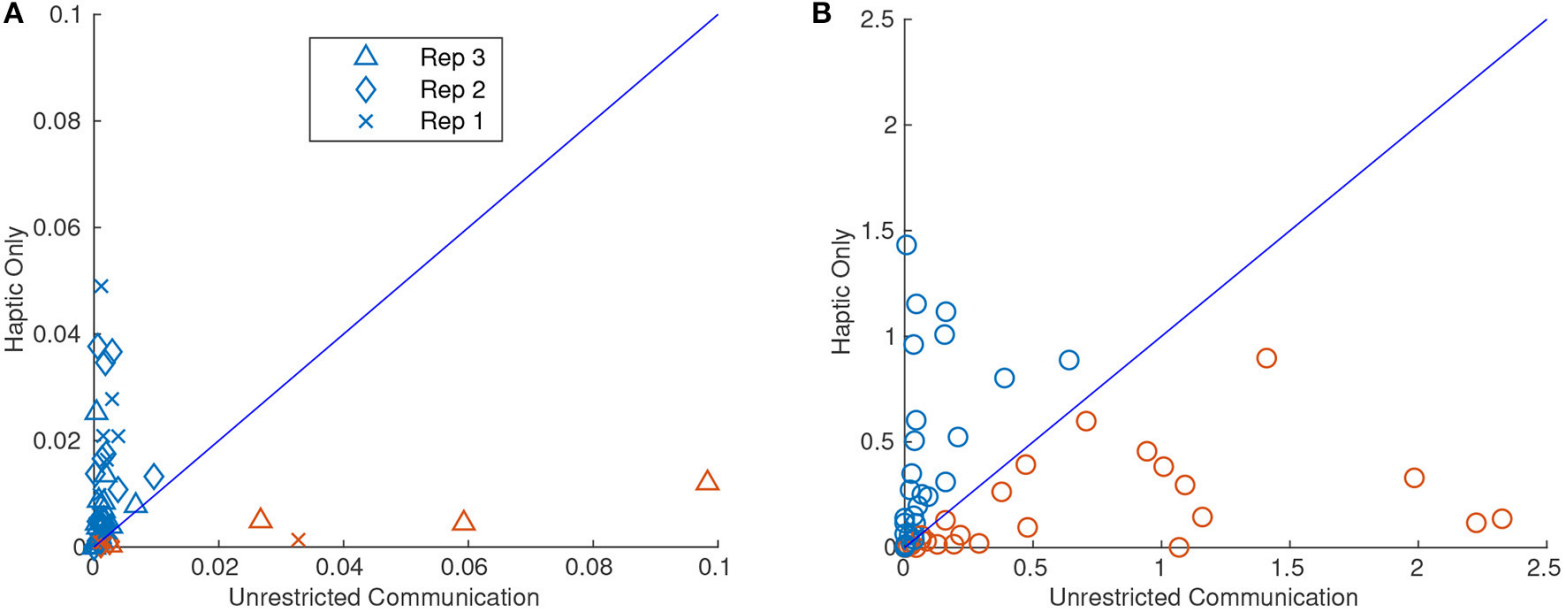
Scatter plots of variance of orientation about Z for the pure translation task **(A)**, and over-rotation about Z for the pure rotation task **(B)**. The X value corresponds to the unrestricted communication (UC) and the Y value corresponds to the haptic-only communication (HC) trials. The blue and red markers indicate an increase and decrease, respectively, in the metric when the dyad completed the task from UC compared to HC. It should be noted in **(A)** that three of the four prominent red markers with high variance were all from the third repetition. If the third repetition is removed, a two-sample *t*-test for equal means rejects the null hypothesis at the 0.01 significance level. In **(B)**, it should be noted that the two groups were not significantly different given the *t*-test above.

Figure 12 shows the same type of scatter plots as Figure 11 but for variance of orientation in Z for the pure translation tasks and area of over-rotation for the pure rotation tasks. As can be seen from Figure 12A, the variance for the HC tasks was usually much higher than their UC counterparts except for four outliers. The “x”s, diamonds, and triangles of the scatter plots represent the 1st, 2nd, and 3rd repetition of the task, respectively. As can be seen from the figure, three of the four large variances for UC were from the third repetition. The large variances seen could then be explained simply by knowing that the dyad had done several repetitions of the task and the leader assumed that the follower knew what they were doing and the leader skipped essential signals. The largest of the three variances can be seen in video format here: https://youtu.be/r96NwCbWDNA. If all of the third repetitions are removed, a *t*-test for equal means between HC and UC variance rejects the null hypothesis at the 0.01 level with a *p*-value of 0.0013.

Figure 12B shows the same type of scatter plot but for the area of over-rotation in the orientation about the Z-direction. This was an interesting result in that there was not a large difference between HC and UC feedback. Indeed, a *t*-test for equal means returned a *p*-value of 0.55. This surprising result could possibly mean that the stopping signal for rotation is primarily haptic in nature and auditory and visual clues are not common communications in stopping a movement. The videos for two of the largest areas of over-rotation for UC and HC, respectively can be found here: https://youtu.be/FvOrp3KF4U4, https://youtu.be/X-r9wNEnk7A. This is good news for pHRI researchers as current control algorithms already implement haptic feedback for all motions.

## 4. Discussion

From this study, valuable insights into the nature of human-human co-manipulation were discovered. Trends were analyzed with respect to completion time, linear velocity, angular velocity, interaction force, and interaction torque. Studying haptic communication was the main focus of this analysis but we also attempted to understand how the quality of the interaction changed when the human dyad was allowed unrestricted (UC) vs. haptic-only (HC) communication, i.e., what happens when the dyad loses visual and auditory communication. The main goal in doing this research is to enable more legible and predictable human-robot co-manipulation.

### 4.1. Insights

We specifically looked at haptic interaction since it is one of the most readily available and widely implemented inputs for pHRI control. We found the following interesting trends and insights with regards to haptic interaction:

- Interaction force and torque (FT), by themselves, are sufficient to complete six DoF co-manipulation tasks, however, there is some loss in the quality of the co-manipulation task (completion time, speed, object placement) if a human-human dyad is restricted to haptic-only communication (HC).
- If the dyad is restricted to HC the dyad suffers from decreased speeds and increased completion time as well as difficulty in maintaining the desired orientation.
- High interaction torque increases when sight and auditory signals disappear, but high interaction force remains largely unchanged.
- High interaction FT values can signal disagreement in speed or proposed path.
- Higher interaction FT values can lead to less confusion in a dyad.
- Interaction torque is more correlated than interaction force to proposed measures of synchrony for lateral translation and superior rotation.
- Acceptable ranges of interaction FT will vary between dyads.

Along with these insights about haptic interaction, we found several more general insights:

- Objects, around the same size and weight as our object, being carried by human-human dyads exhibit an oscillation of approximately 1.33–1.66 Hz. This is most likely a result of human gait and the resulting dynamic motion of the dyad and object together.
- Intentional movement of the carried object usually occurs below 0.66 Hz and mostly below 0.33 Hz which is consistent with current research.
- Median velocity and completion time have a strong, negative, and nonlinear relationship.
- Human-human dyads were able to perform faster and had shorter completion times as they became familiar with the tasks.

### 4.2. Possible Applications to pHRI

We propose that these insights could be applied to pHRI in many ways. As shown in section 3.1, the learning process experienced by the dyads will definitely need to carry over to pHRI. Engineers that design robot hardware and controllers must be aware that humans will need time to adapt to a robot controller even if it is programmed to behave exactly like an average or nominal human. Changes to the range of interaction forces and torques, and average speeds will most likely be necessary as the human continues to work with the robot. We expect that humans will generally be willing to perform a task faster if they have already completed a similar task with the robot.

As seen in section 3.2, pHRI controllers could transform high interaction torque to a measure of a lack of synchrony for translational movements and possibly for rotational movements as well. Robots could monitor interaction torque to signal a change in speed or direction parameters. Human-robot co-manipulation control algorithms could be successful at improving synchrony by seeking to control interaction torque, perhaps requiring only haptic sensors as opposed to more complicated visual and auditory sensing and semantic interpretation.

We also found that interaction force seemed to correlate with angular velocity. This trend could apply to pHRI in many different ways. For example, if the interaction force begins to drop, the robot could begin a rotation movement. Also this trend could be applied by the robot intentionally dropping the interaction force to try and speed up the rotational movement or increasing interaction force to decrease rotational movement. Additionally, a pHRI control law designed for translation tasks could therefore maintain low interaction torques to keep angular velocity down.

In section 3.3, we found that the object being carried by human-human dyads tends to exhibit a periodic motion. Humans may prefer this type of oscillatory motion because of previous experience and thus this motion may need to be included in a human-robot co-manipulation controller if the objective is legible and predictable motion. This could be included by making the robot aware of this motion and recognizing that it may not be a form of communication or it could be included by the robot looking to match that same oscillatory motion. The latter option may help the interaction to feel more natural to the human and help the human feel better about the interaction as a whole. Sylos-Labini et al. (2018) found that people walking side by side often synchronized their footsteps and hypothesized that interaction forces may have helped them come into synchrony. Similarly, this oscillatory motion may have helped the dyad come into sync with each other which would then improve the overall quality of the interaction.

Lastly, in section 3.4, we found that there is a significant difference in interaction torque and speed when restricted to haptic feedback only. These trends are important to pHRI since all communication between humans and robots in physical tasks is happening haptically as of the current literature. The applicability of this to pHRI might be suspect due to the difference in human and robot capabilities. However, a successful controller may need to change control strategies if visual or auditory feedback to the human is being hampered by such things as lifting a large item or working in a noisy environment. The robot could be programmed such that it can recognize that these obstructions are occurring and then slow down and increase interaction torques for acceptable co-manipulation. The robot might also change its estimation of completion time if that becomes important for path-planning or other control operations. Additionally, the robot must recognize that the human will not be very good at maintaining a static orientation and the robot may have to compensate for this effect.

We assume that these trends and insights will help robotics researchers program better partner robots. However, that is not the only goal with these insights. In the future, robots could be tasked with trying to persuade the human into moving faster or taking a different route. This is especially true for several reasons: (1) given that robots can be equipped with many different kinds of sensors that are at times superior to human eyesight, (2) given their ability to gather and process information from outside sources such as GPS data, and (3) the ability to monitor vital signs of a patient in a search and rescue scenario. If a robot were tasked with leading a human follower then these trends in the data may be even more important to exploit and develop.

### 4.3. Limitations of the Scope of This Research and Future Work

The scope of this paper does not include proof of pHRI application but only observations of HHI and speculations as to the applicability in pHRI. Thus, the most natural next step from this work is to create and evaluate a human-robot co-manipulation controller that incorporates some of the insights found. Many of the insights can be implemented as general guidelines for developing pHRI controllers although some of the insights we found could also evaluate the similarity of human-robot interaction and human-human interaction.

We studied what we deem as nominal behavior for a human-human dyad and future research will need to focus on how human-human dyad behavior changes when external modifiers are added to the environment. This could be done by studying what happens to the behavior of the dyad when the task must be done quickly or quietly. We assume that some of the insights we found are solely based on nominal human performance and do not carry over to specified objectives. However, we still expect some insights to generalize to all human behavior.

### 4.4. Conclusion

In conclusion, we suggest that the insights contained within this paper will help pHRI researchers to develop and evaluate human-robot co-manipulation controllers with specific regard to the legibility and predictability of those controllers. We suggest that interaction torque will be a large part of future pHRI co-manipulation studies and may be a readily available metric for evaluating the quality of the dyadic relationship involving robots.

## Data Availability Statement

The raw data supporting the conclusions of this article are available at the following 10.6084/m9.figshare.13669178.

## Ethics Statement

The studies involving human participants were reviewed and approved by Brigham Young University Institutional Review Board. The participants provided their written informed consent to participate in this study. Written informed consent was obtained from the individual(s) for the publication of any potentially identifiable images or data included in this article.

## Author Contributions

MK designed and oversaw implementation of the experiment. SJ, JS, and MK analyzed the results. SJ wrote the main manuscript and prepared figures with significant edits by JS and MK. All authors reviewed the manuscript.

## Conflict of Interest

The authors declare that the research was conducted in the absence of any commercial or financial relationships that could be construed as a potential conflict of interest.
